# Metal nanoclusters: from fundamental aspects to electronic properties and optical applications

**DOI:** 10.1080/14686996.2023.2222546

**Published:** 2023-06-21

**Authors:** Rodophe Antoine, Michel Broyer, Philippe Dugourd

**Affiliations:** Univ Lyon, Univ Claude Bernard Lyon 1, CNRS, Institut Lumière Matière, Villeurbanne, France

**Keywords:** Metal cluster, Nanocluster, noble metal, magic numbers, plasmon, superatom, ligand-stabilized nanocluster

## Abstract

Monolayer-protected noble metal clusters, also called nanoclusters, can be produced with the atomic precision and in large-scale quantity and are playing an increasingly important role in the field of nanoscience. To outline the origin and the perspectives of this new field, we overview the main results obtained on free metal clusters produced in gas phase including mainly electronic properties, the giant atom concept, the optical properties, briefly the role of the metal atom (alkali, divalent, noble metal) and finally the atomic structure of clusters. We also discuss the limitations of the free clusters. Then, we describe the field of monolayer-protected metal clusters, the main results, the new offered perspectives, the added complexity, and the role of the ligand beyond the superatom concept.

## GRAPHICAL ABSTRACT

**Figure:**
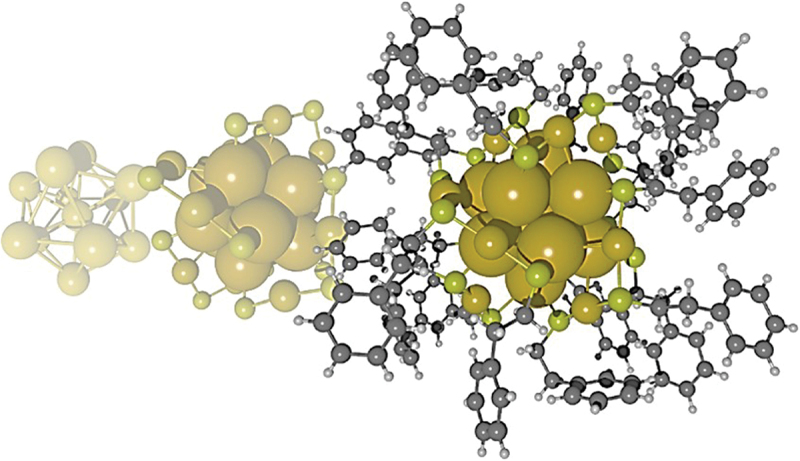

## 1. Introduction

The fascinating properties of small metallic particles have progressively emerged since the antiquity. In middle age, the glaziers discovered how to produce beautiful glasses with metallic inclusions. In 1856, Faraday made a huge step in the understanding of these phenomena [1,2]. He showed that the optical properties of gold colloids were different from those of thin gold leaves [3], thereby launching the field of nanoscience. Then, the exceptional optical properties of small metallic particles were explained in detail by Rayleigh, Mie and others through electromagnetism, founding the light matter interaction. Other important properties were then discovered for small particles. For example, Buffat and Borel [4] showed in 1976 that the melting temperature of gold nanoparticles depends on their sizes and decreases drastically for sizes around 2 nm diameter. Very similar results were reported earlier [5–8]. These new properties were not limited to these two examples and it became necessary to revisit condensed matter science for small particles leading them progressively to the emerging of nanoscience.

However, in 1960s and 1970s, nanoparticles were produced with dispersed and poorly characterized sizes; they were most often deposited on supports or synthesized in colloids. It was difficult to separate the specific properties of these small particles from the interaction with the support and more generally with the particle environment. Then in the late 1970s and during the 1980s, with the development of supersonic molecular beams, it became possible to produce free clusters M_n_, M being a metallic atom and n the number of atoms varying from few units to few hundred or few thousand depending on beam conditions.

**Figure 1.**
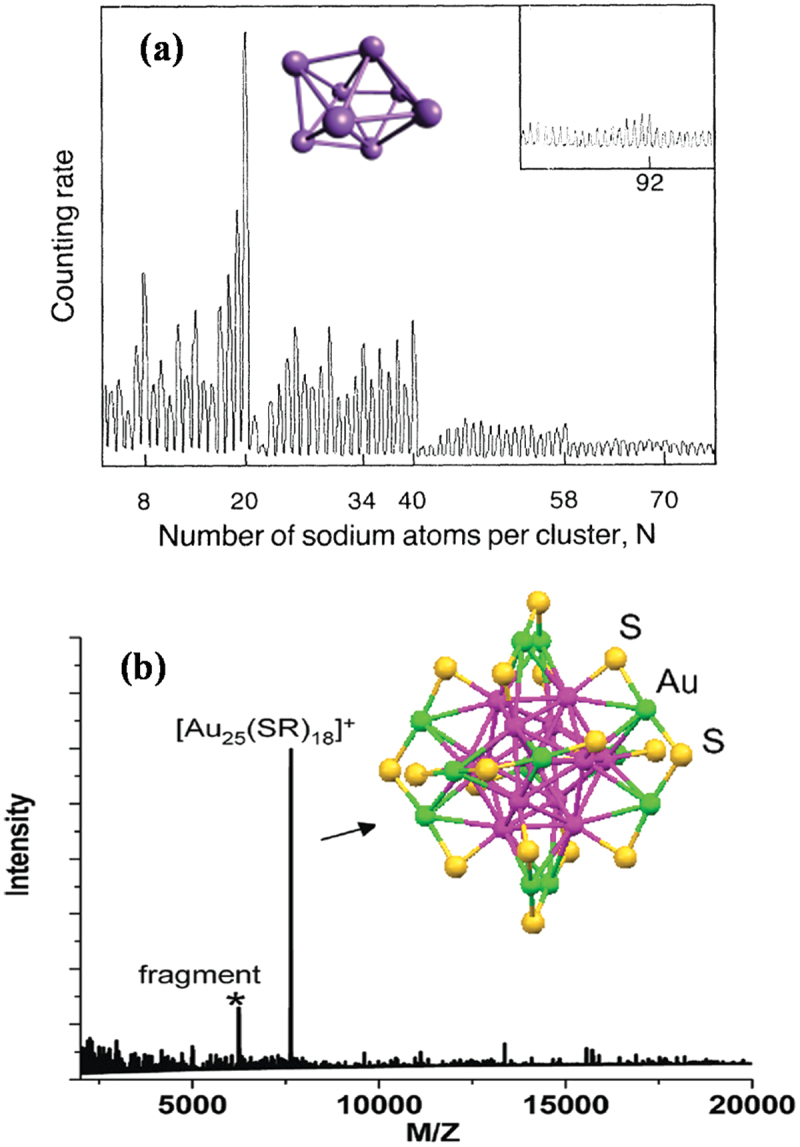
(a) Mass spectrum of sodium clusters, *N* = 4–100 (including insert), and optimized Na_8_ clusters in inset. Reproduced with permission from ref [10]. American Physical Society, copyright 1984. (b) Matrix-assisted laser desorption time-of-flight mass spectra of the Au_25_ nanoclusters protected by chirally modified phenylethylthiolate ligands (SR), and the corresponding crystal structures of Au_25_SR_18_ in inset. Reproduced from ref [12]. with permission from American Chemical Society, copyright 2011.

The alkali clusters were then produced and controlled in size by mass spectrometry [9–11]. Dramatic size effects were observed with best stabilities for well-defined number of atoms called magic numbers (Figure 1(a)). They were interpreted in the frame of an electronic shell model, associated with the spherical symmetry of the effective potential experienced by each delocalized electron [13]. At about the same time, Smalley and collaborators developed a laser vaporization source able to produce free clusters of refractory metals, in particular transition metal cluster [14].

These new experimental beam techniques, associated with mass spectrometry and laser spectroscopy, paved the way to the study of electronic, optical, magnetic, catalytic properties of isolated clusters as a function of sizes. Two main results were obtained, first a general trend for an evolution as a function of radius R of the cluster, and secondly specific effects observed for well-defined number of atoms (each atom counts). However, if molecular beams are well-fitted for the detailed study, in gas phase, of clusters, the produced clusters are very fragile and available in very small quantities. They are also very difficult to deposit on surfaces or in matrices and then to characterize at solid state level, for example by X-ray diffraction. In that sense, the discovery of the fullerene C_60_ was instructive: in 1985, Kroto, Smalley et al. observed an intense peak in mass spectra corresponding to C_60_ [15]. They showed by varying beam conditions that this molecule was really stable. They proposed the now well-known and elegant cage structure of icosahedral symmetry (Buckminsterfullerene). But they did not succeed to synthesize a sufficient amount of C_60_ by molecular beam and mass spectrometry techniques. Finally in 1991, Krätschmer et al. [16] synthesized a solid fullerite made by the crystal arrangement of C_60_, by condensing graphite vapor from an electric arc in an inert gas. The obtained powder was then purified by solvents, and the structure proposed by Smalley et al. was confirmed by X-ray diffraction. But the example of C_60_ (and of a few other carbon fullerenes) was unique and could not be generalized to metal clusters such as Au_8_ or others, in particular due to their high reactivity.

Following the pioneering route started by Brust and colleagues [17], Whetten et al proposed and succeeded in a seminal paper to isolate gold nanocrystals (nano-scaled crystallites) [18]. The building blocks of gold clusters are passivated by self-assembled monolayers of straight-chain alkylthiolate molecules [19]. This paper and all results obtained in the following years, opened a new field called nanoclusters. Clearly it became possible of producing well-defined protected metal clusters, at the atomic precision, meaning that nanoclusters with a specific number of n metal atoms and m thiolated ligands (SR) can be produced [20,21]. As a result, mass spectra are only composed by one main peak corresponding to the nanoclusters obtained at the atomic precision as illustrated for Au_25_(SR)_18_ in Figure 1(b)) [12]. This unique feature mainly arises from the fact that the well-defined Au_25_(SR)_18_-protected metal clusters [22] are electronically stabilized by a metal core composed by eight confined electrons (as detailed in section III) [23]. This transition from magic numbers of electronic shell model of free clusters to protected metal cores illustrates the change of paradigm between the free clusters and protected nanoclusters [24].

The purpose of the present paper is to describe the emergence of nanoclusters and their important role in the field of nanoscience. To situate the origin and the perspectives of this new field, we recall the main results obtained on free clusters, including mainly electronic properties, the giant atom concept, the optical properties, briefly the role of the metal atom (alkali, divalent, noble metal) and finally the atomic structure of clusters. We discussed also the limitations of the free clusters. Then, we described the field of monolayer-protected metal clusters, the main results, the new offered perspectives and also the added complexity, the role of the ligand beyond the super atom concept. Finally, we discuss the linear and nonlinear optical properties of nanoclusters, as well as future perspectives.

## 2. Free metal clusters

### 2.1. Electronic shell model

Figure 1(a) shows that neutral sodium metal clusters have best stabilities for magic numbers 8, 20, 40, 58, 92 etc [10,25]. This remarkable result was interpreted by an electronic shell model very similar to the nuclear shell model or the periodic table for atoms [25]. In these three cases, the remarkable stabilities for magic numbers are in close relationship to the spherical symmetry of the effective potential experienced by the electrons.

At first approximation, a cluster of N sodium (or metal) atoms may be considered as a sphere of radius *R* = r_s_N^1/3^ where r_s_ is the Wigner Seitz radius. Different simple spherical potentials have been used for clusters, the square-well potential, the harmonic potential, and the Woods-Saxon potential:

(1) $$U\left(r\right)=-\frac{U_{0}}{\exp\left[\frac{r-R}{a}\right]+1}$$

where U_0_ is the sum of the Fermi energy and of the work function for the bulk (respectively 3.23 eV and 2.7 eV for sodium). The parameter a is typically 1.5 a.u. for sodium.

**Figure 2.**
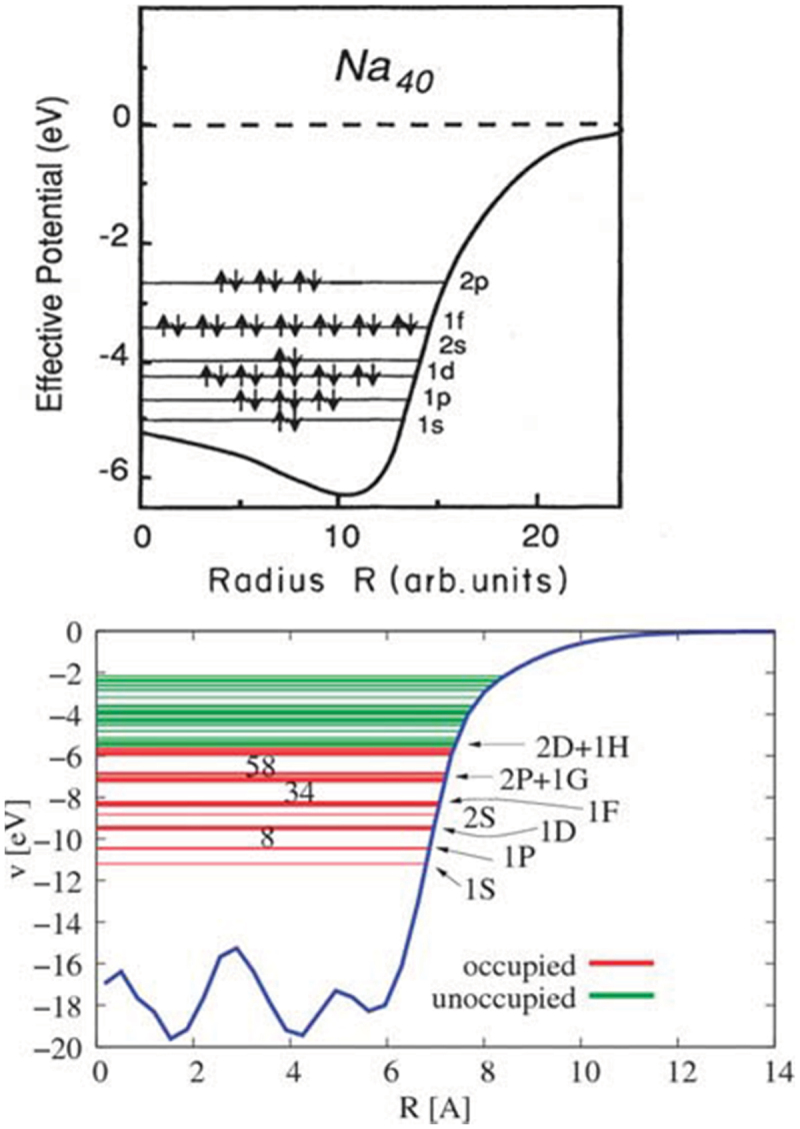
Top: electron gas model for Na_40_ cluster assuming a spherical ‘jellium’ background potential. Bottom: DFT calculation for the Au_79_ core of Au_102_(p-MBA)_44_ shows the shell structure of the core. Reproduced by permission from ref [26], Copyright 2016 SPIE digital library.

A better approximation is given by the jellium model. The electrons are assumed delocalized in a sphere R of positive charge of constant density, such as the global neutrality of the cluster is obtained. The energy of this system is then self-consistently calculated and the effective potential experienced by electrons may be obtained. Figure 2 shows examples of electronic potentials for metal clusters in the jellium model. Amazingly, one can draw a parallel with electronic properties of metal kernels in monolayer-protected nanoclusters. Indeed, the model calculation for the bare Au_79_ core just considering Au to be 6s-monovalent metal (see Figure 2 (bottom) showing the DFT calculation for the Au_79_ core of Au_102_(SR)_44_ evidencing the shell structure of the core), compared well with the electronic potentials obtained from the classic case of gas-phase sodium clusters [25].

If we consider the evolution of self-consistent potential when the size increases, the shape of the surface profile, the bottom value, and the Fermi level are quasi-independent on the size. We obtained a flat-bottomed potential with the same surface profile almost independent on N [27]. It is interesting to remark that a large variety of similar spherical potentials give roughly the same magic numbers: Wood-Saxon, harmonic, hard square potentials [25] and the two potentials of Figure 2. But this does not mean that all properties of these ‘different fictive clusters’ are identical.

The spherical approximation for metal clusters is valid only for non-degenerate states, as electronic closed shells. But for open-shell clusters, the electronic states are degenerate, and clusters distort as stated by the Jahn-Teller theorem. The degenerate levels are split. For small size such as Na_8_, the gap between the highest occupied and lowest unoccupied molecular orbitals (HOMO-LUMO) is large, typically of the order of 1 eV, but for open shells clusters, this gap is reduced by the splitting of the levels. A model of ellipsoidal deformation of clusters has been proposed by Clemenger [28] in analogy with the Nilsson model for nuclei. This model assumes an effective harmonic potential. The ellipsoidal deformation may be calculated for small clusters and agree with experimental mass spectra [28]. The spherical symmetry refers to the metal droplet model. Such model is reasonable if the produced clusters in beams have temperatures close to their melting points. This is often the case for alkali clusters partly because alkali metals have low melting point and partly because in most cases, the melting point decreases when the size decreases. If the clusters are faceted, the degeneracy of energy levels is reduced. In the limit of Kubo [29], due to irregular faceting, the levels are no more degenerate. Kubo assumed in first approximation that the electronic states are not degenerated (except the factor 2 due to the electron spin) and that the energy gaps are all equal. Assuming for simplicity a square-well potential, this energy splitting δ is then given by the formula:

(2) $$\delta =\frac{4E_{F}}{3N}$$

This rough approximation results in a very simple formula which represents the inferior limit for the averaged energy gap in clusters. In practice, the energy gaps are slightly larger in free metal clusters. If we use the Kubo formula, the gap for a cluster Na_N_ with *N* = 100 is 43 meV and corresponds to about *T* = 500 K.

As it will be explained below, nanoclusters are encapsulated in ligands and have much more rigid structures. The symmetry is generally preserved resulting in larger gaps [21]. As we will see, this has important consequences on the observation of fluorescence from the optical excitation of the free clusters and nanoclusters. Before discussing this point, let us present the optical spectroscopy of free clusters.

### 2.2. Spectroscopy of free alkali clusters

Absorption spectroscopy of neutral free clusters in molecular beams is difficult to observe directly from the extinction of light, because the cluster beam contains many sizes of low density. Therefore, absorption [30] and fluorescence [31] spectroscopies have been reduced to a few examples. The optical spectroscopy is associated with mass spectroscopy via two photon ionization or depletion spectroscopy [32]. In fact, in most cases, due to relatively low binding energies, the absorption of visible or UV light by free alkali clusters is followed by dissociation or photo-evaporation of atoms. The fragments are then ejected from the beam and are no more detected by mass spectroscopy at the extremity of the beam. The parent clusters are then depleted.

**Figure 3.**
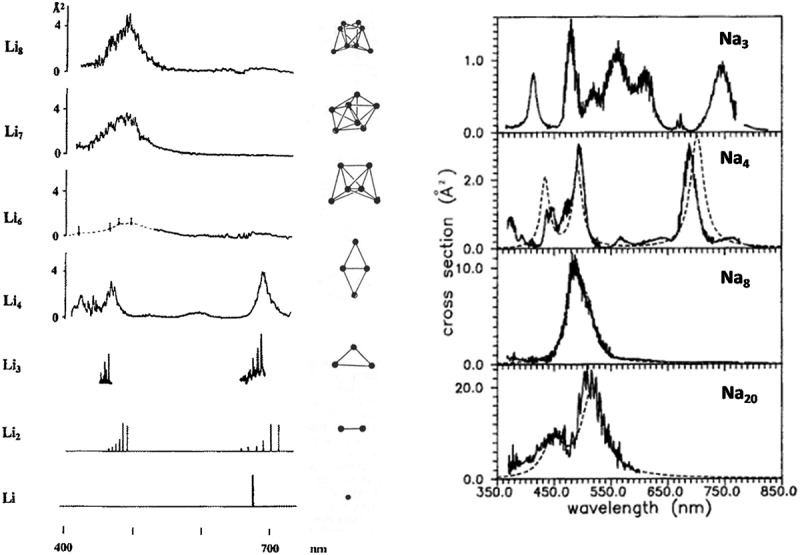
(Left) Absorption spectra of Li_n_ clusters *n* = 1–8. The results for n≤3 were recorded by means of resonant two-photon ionization, while the results for *n* > 3 were obtained by means of depletion spectroscopy. The corresponding geometries result from the comparison of the spectra with CI ab initio calculations. Reproduced by permission from ref [35], Copyright © by R. Oldenbourg Verlag, München 1996. (right) Absorption spectra of Na_n_ clusters *n* = 3, 4, 8 and *n* = 20, obtained by depletion spectroscopy. Reproduced by permission from ref [36] Copyright © by Springer-Verlag 1991.

For very small clusters, rich transitions of molecular type are observed with vibrational and rotational structures in small alkali clusters Na_3_ and Li_3_ and Li_4_ [33,34]. Figure 3 shows examples of absorption spectra of Li_N_ and Na_N_ clusters [35–38]. When the size increases, the spectra tend to be reduced to one or two broad resonances (Li_7_, Li_8_, Na_8_, Na_20_) [37,38]. These broad resonances are the premises of the surface plasmon resonance (Mie resonance) corresponding to the oscillations of the electron cloud as compared to the ionic background of the metal clusters.

In classical electromagnetism, the Mie resonance may easily be calculated in the dipolar or quasi-static approximation. It means that the variation of light electric field on the dimension of the nanoparticle is neglected. It corresponds to R << λ where λ is the wavelength of the light. This approximation is valid for small clusters (R<10nm) in the optical domain (λ∼400−700nm).

In the framework of this approximation, the response of a metallic particle to an optical wave ω may be obtained from the dielectric constant of the metal ε(ω) = ε_1_ + iε_2_ where ε_1_ (ε_2_) is the real (imaginary) part of this dielectric constant. In the ‘free electron’ model mainly valid for alkali metal or Drude model, ε_1_ and ε_2_ are given by:

(3) $$\varepsilon_{1}\left(\omega \right)=1\;-\;\frac{\omega_{p}^{2}}{\omega^{2}+\Gamma^{2}}=\;1\;-\;\frac{\omega_{p}^{2}}{\omega^{2}}$$

(if Γ, the damping constant, is small as compared to ω in the optical domain)

(4) $$\varepsilon_{2}\left(\omega \right)=\;\frac{\omega_{p}^{2}\Gamma }{\omega^{3}}$$

The absorption cross-section σ(ω) by the cluster of radius R in a medium of permittivity ε_r_ is:

(5) $$\sigma \left(\omega \right)=\;\frac{9\omega \varepsilon_{r}^{3/2}}{c}\;\frac{4\pi R^{3}}{3}\;\frac{\varepsilon_{2}}{{\left(\varepsilon_{1}+2\varepsilon_{r}\right)}^{2}+{\left(\varepsilon_{2}\right)}^{2}}$$

where c is the light velocity and Γ the damping constant.

A resonance is obtained for ε_1_(ω) + 2ε_r_ = 0

It is called the surface plasmon resonance ω_sp_ (or Mie resonance) and is given by:

(6) $$\omega_{sp}=\frac{\omega_{p}}{\sqrt{1+2\varepsilon_{r}}}$$

where ω_p_ is the volume plasmon pulsation, $\omega_{p}^{2}=\frac{n_{e}q_{e}^{2}}{m_{e}\varepsilon_{0}}$, n_e_ being the electronic density in the cluster, q_e_ the electron charge and m_e_ the electron mass.

The absorption cross-section σ(ω) is then proportional to

(7) $$\sigma \left(\omega \right)∼\frac{R^{3}\varepsilon_{r}^{3/2}\Gamma \omega^{2}\omega_{sp}^{2}}{c\left(1+2\varepsilon_{r}\right)\left[{\left(\omega^{2}-\omega_{sp}^{2}\right)}^{2}+\frac{\omega_{sp}^{4}\Gamma^{2}}{\omega^{2}}\right]}$$

In the classical limit, the resonance is a quasi-Lorentzian curve at first approximation: if we write ω ~ ω_sp_ in the numerator and in the damping term of the denominator, we obtain:

(8) $$\sigma \left(\omega \right)∼\frac{R^{3}\varepsilon_{r}^{3/2}\Gamma \omega_{sp}^{2}}{4c\left(1+2\varepsilon_{r}\right)[{\left(\omega -\omega_{ps}\right)}^{2}+\left(\Gamma /2{)}^{2}\right]}$$

**Figure 4.**
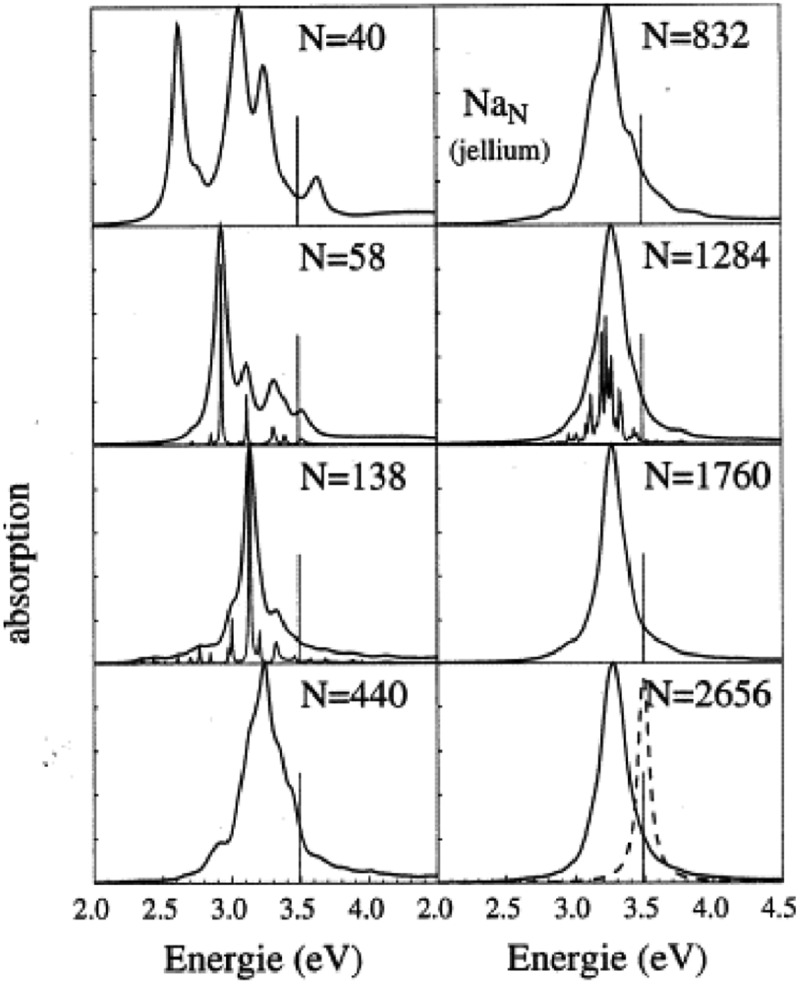
Photo-absorption cross-sections for sodium clusters (Na_N_) obtained under the TDLDA formalism in the frame of the jellium model ref [27]. The values of N correspond to complete electronic shells (spherical clusters). In the calculations the natural width of individual level is 0.1 eV. For *N*= 58, 138 and 1284, calculations are also done with an arbitrary width of 0.01 eV, in order to illustrate the fragmentation of the resonance (curves below with fine resonances). The dashed curve corresponds to the resonance in the limit of very high value of N. Copyright © Belin.

For sodium, using the electronic density of the bulk, we obtained for the resonance ω_sp_ = 3.49 eV from formula (6) with ε_r_ = 1. For small clusters, the resonance is red-shifted because of the spill out of electrons. The radius of small clusters is in fact R+δ where R is calculated from the bulk density n_e_ and δ the spill out. For the given number N of atoms, R = r_s_N^1/3^, r_s_ being the Wigner Seitz radius, occupied by one atom (corresponding to one electron in alkali). Taking into account this spill out, the resonance becomes ω_sp_(R) = ω_sp_ (1–3δ/2 R) = ω_sp_ (1–3δ/(2 r_s_N^1/3^)). With r_s_ = 2.1Å and δ = 0.69Å, we find ω_sp_(Na8) = 2.63 eV. It is not so far from the experimental peak of the absorption of Na_8_ which is 2.55 eV (486 nm), if we consider the approximations made to estimate the resonance peak. The evolution of resonance as function of the size in Na_N_ clusters is discussed in refs [27,39]. For lithium clusters, the interpretation is similar but more complicated due to an effective mass for electrons in bulk lithium [31]. The spectra of Figure 3 may be theoretically calculated by ab initio quantum chemistry [40] where the positions of each atom is taken into account. These calculations well reproduce the experimental spectra. In fact, for larger clusters (N > 20), TDLDA (Time Dependent Local Density Approximation) calculations performed in the frame of the jellium model illustrates how appear the collective excitation of electrons (Figure 4). The vertical bar in Figure 4 corresponds to the surface plasmon resonance ω_sp_ = 3.49 eV, obtained from formula (6). We see that globally the resonances are red-shifted due to spill out (see discussion above) and even for N = 2656 (R = 2.9 nm) [21,39] the spill out has a non-negligible influence. The collective state interacts with the mono-electronic excitations (called particle-hole excitations) and this results in the fragmentation of the resonance (Figure 4). At large size, the fragmentation of the resonance is responsible for the width Γ of the resonance or Landau damping. The existence of at least two peaks in the experimental spectrum of Na_20_ may be interpreted as the fragmentation of the resonance. However, Na_20_ may be calculated by ab initio quantum chemistry including the positions of all atoms [40] while the spectra of Figure 4 are model calculations in the frame of the jellium model.

### 2.3. Spectroscopy of noble metal clusters

The alkali metals (and earth alkali) follow roughly the Drude theory for the dielectric constant ε(ω) = ε_1_ + iε_2_ with ε_1_ and ε_2_ given by Equations (3) and (4).

For transition metal in general, the dielectric constant does not follow the Drude formulas (3) and (4) because of interband transitions from d electrons. The contributions of s and d electrons are mixed. And most of the transition metals with incomplete d bands have no clear Mie resonances [41].

The situation is different for noble metal (Cu, Ag and Au) which have a complete d band (d^10^ s^1^ as atomic structure). It is then possible to separate the contribution of s and d electrons in the dielectric constant, respectively, ε^s^ and ε^d^. If the susceptibility $\chi_{s}\;and\;\chi_{d}$of, respectively, s and d electrons are introduced, the dielectric constant ε(ω) of noble metals may then be written as:

(9) $$\begin{array}{rl} & \varepsilon (\omega \;)\;\;=\;\;1\;+\;\chi_{s}\;+\;\chi_{d}\;=\;\varepsilon {\;}^{s}(\omega \;)\;+\;\varepsilon {\;}^{d}(\omega \;)\;-\;1\;\;\\ & \;\;\;\;\;\;\;\;\;\;=\;\;\varepsilon {{\;}^{s}}_{1}(\omega \;)\;\;-\;\;1\;+\;\varepsilon {{\;}^{d}}_{1}(\omega \;)\;\;+\;\;i\;(\varepsilon {{\;}^{s}}_{2}(\omega \;)\;\;+\;\varepsilon {{\;}^{d}}_{2}(\omega \;)) \end{array}$$

Then,

(10) $$\varepsilon_{1}\left(\omega \;\right)={\varepsilon^{d}}_{1}\left(\omega \;\right)-\frac{\omega_{p}^{2}}{\omega^{2}}$$

(11) $$\varepsilon_{2}\left(\omega \;\right)\;={\varepsilon^{d}}_{2}\left(\omega \;\right)+\frac{\omega_{p}^{2}\Gamma }{\omega^{3}}$$

ε^d^_1_(ω) and ε^d^_2_(ω) are obtained from the measured dielectric constants, the Drude dielectric function and a Kramers-Kronig analysis.

The formula (6) for the surface plasmon resonance (SPR) becomes

(12) $$\omega_{sp}*=\frac{\omega_{p}}{\sqrt{\varepsilon_{1}^{d}\left(\omega_{sp}^{*}\right)+2\varepsilon_{r}}}$$

Since ε^d^_1_(ω) depends on ω in a non-analytic form, the solution is graphic. In the spectral domain of the SPR, ε^d^_1_(ω) is typically 4–5 for Ag and 10 for gold. This explains why in vacuum (ε_r_ = 1) the SPR of noble metal is in the visible region (typically 3.5 eV for Ag and 2–2.5 eV for gold and copper) while ω_p_ the volume plasmon calculated by the formula $\omega_{p}^{2}=\frac{n_{e}q_{e}^{2}}{m_{e}\varepsilon_{0}}$ is around 9 eV [42].

For silver, the threshold for interband transitions is 4 eV (ε_2_^d^(ω) = 0 for ω < 4 eV). Therefore, the SPR is well separated from the interband transitions and the situation is similar to that of alkali clusters. The main difference is the shift when the size decreases. In alkali clusters, the SPR is red-shifted because the spill out of electrons tends to decrease the electronic density n_e_ and consequently to decrease ω_p_. In noble metals, the screening due to ε^d^_1_(ω) is inefficient close to the surface, this effect tends to increase the value of ω_sp_* and compensates the spill out. The tendency for noble clusters is rather a blue shift when the size decreases [42].

For gold and copper clusters in vacuum, the value of ω_sp_* is very close and even above to the interband threshold. Therefore, the SPR emerges very difficult for small sizes, there are many particle-hole excitations involving s and d levels, as illustrated by the spectroscopy of monodispersed Cu_n_ and Au_n_ clusters [43,44] deposited in rare gas matrices with 1≤n≤9. The absorption curves show many optical transitions and are very different from the curves for Na_n_ and Li_n_ (Figure 3) where few intense transitions dominate.

For copper and gold clusters, the SPR emerges typically from clusters of more than 150–200 atoms [43,45]. The reason is the mixing of transitions from s and d levels. When the size increases, the s and d bands develop. Moreover, the SPR tends to shift to the red when the size increases and emerges on the red side of the interband transitions. If the clusters are embedded in a medium ε_r_, the resulting red shift (formula (12)) fosters the emergence of the SPR [42] because the threshold of inter-band transition does not shift with ε_r_ and the surface plasmon resonance no more interacts with inter-band transitions.

A non-metal/metal transition in copper or gold is sometimes associated with the emergence of the SPR [46]. But it is not clear that the plasmon may be a signature of the metallic character. As already noted, most of transition metals have no clear plasmon resonance, nevertheless they are good conductor for electricity. Moreover, a clear volume plasmon is observed in silicon bulk [47] and also a SPR in silicon clusters [48].

The metallic character of small metal clusters may be discussed on a different points of view. The first aspect is to consider the electrons responsible of the bonding. In covalent systems, the electrons roughly localized on the line connecting two atoms. In ionic bonding, the electrons are located on the atoms. In metals, the electrons are delocalized, this means that they are between the atoms. In this respect in Na_5_ and even in Na_3_ [40,49], the electrons are already delocalized and the bonding is metallic. It is similar for transition metal clusters even if the bonding is more complicated with both d and s electrons. The only exception is divalent metal clusters which have an ns^2^ atomic structure. The metallic character comes from the mixing of s and p band which occurs as the size increases from typically 10 or 20 atoms, except for mercury where the gap closure between s and p bands occur around 200 atoms [50]. Therefore, for most divalent metal clusters, the metallic character of the bonding is present for very small sizes even if the progressive building up of the metallic bands needs a given number of atoms depending of studied element.

While the characterization of a metallic material is clear in the case of bulk systems, where band theory concepts apply, this concept is more subtle for small clusters [51]. We may consider also the conduction of electrons. Indeed, in this case, we know that the electronic gap must be smaller than kT. As discussed above (see the Kubo formula, Equation (2)), at room temperature, small metallic clusters of less than 100 atoms are semiconductors and it is also true for larger clusters of high symmetry. This property does not prevent the SPR to emerge despite the fragmentation of the resonance.

In conclusion, the emergence of the SPR is not really pertinent to probe the metallic character. The photo-electron spectroscopy (photo-emission in solid physics) is the best way to observe the building up of electronic bands as the size increases [52,53]. The occurrence of metallic conductivity is a complex phenomenon which depends of the atomic structure of elements, of the temperature, of the symmetry. If the cluster is large enough and if the electrons are delocalized, metallicity occurs. We believe that the analysis of molecular orbitals in small clusters is the best way to understand the emergence of metallic character, even if it is more complex than the occurring of a resonance on an optical spectrum. In fact, the SPR is a collective excitation corresponding to interferences of mono-electronic excitations, only recognizable, in ambiguous cases, by a quantum analysis of eigen functions. The macroscopic signature of the SPR is the proportionality of the absorption cross-section to the cluster volume (formula (7) and (8)), but for probing it implies to vary the radius R.

In conclusion of this part, the free metal clusters have exceptional properties, in particular in optics. But they are very fragile and easily oxidize. The fluorescence is difficult to observe in clusters beams, due to small density of particles and to competition with direct dissociation or evaporation of atoms after light absorption. The latter were used to record optical spectra of clusters in molecular beams. For example, the fluorescence of Ag_8_ has been observed [54], but at low temperature, embedded in gas argon matrix or in helium droplets, the argon or helium preventing the dissociation. These are very nice experiments but not adapted for applications. In the second part of this paper, properties of clusters protected by ligands are discussed, as well as the influence of ligands.

## 3. Adding complexity: thiolate-protected metal clusters. From superatom concept and beyond

Free or bare metal clusters (described in the previous sections) are typically unstable in condensed phase (solid and solution), which limit their applications. Solid gas or inorganic matrices can be used allowing for protection of metal clusters against degradation and in particular oxidation or photodissociation [55–59]. But such templates are used as matrices which do not chemically interact with clusters, limiting possible synergetic ‘host-guest’ effects. Thiols are frequently used on noble metal substrates because of the strong affinity of sulfur for these metals, leading to a covalent anchoring with metal-sulfur bond. And thiolate ligands (−SR) have appeared to be extremely good candidates to produce ultrasmall nanocluster sizes, in particular for gold [21]. Following the pioneering work of Brust et al. based on the reduction of the metal precursors and the formation of metal core, thiol-containing small molecules were extensively used to stabilize gold nanoclusters (AuNCs) in the aqueous solution [17]. The use of thiol-containing small molecules as stabilizers permits to better control the production of AuNCs than phosphine-capped ones, contributing to the stronger Au-S covalent bonding. On the other hand, other organic scaffolds allow the formation and stabilization of metal clusters in solution as pioneered by the group of Dickson in 2002 for encapsulating silver nanoclusters with dendrimers or DNAs templates [60–64]. These organic scaffolds have tremendous potentials, as the interaction between the ligands and metal clusters can be adjusted. By playing with the nucleotide sequence of DNA oligomers for instance, it is possible to synthesize silver nanoclusters that emit from the blue to near-infrared region [65]. However, with such scaffolds, the control of the size of nanoclusters (at the atomic precision) is still challenging. Generally, the method of synthesizing thiolate-capped AuNCs has processes as follows. Gold salts [AuCl_4_]^−^ are dissolved in water and then transferred to an organic solvent by phase transfer agent; the thiols are added to the mixture inducing reduction of Au^3+^ ions into Au^+^ ions and form Au^+^-SR complexes or polymers; then the Au^+^ polymers are reduced by adding the reducing agent and lead to thiolate-protected gold nanoclusters. Whetten and coworkers have proposed an unprecedented thiolate-protected AuNC route using the GSH (*N*-γ-glutamyl-cysteinyl-glycine) as the stabilizer. The as-synthesized AuNCs were fractionated by using polyacrylamide gel electrophoresis (PAGE) and characterized by mass spectrometry (MS) [66]. Such nanoclusters have a general formula Au_n_SR_m_, exhibiting (n-m) bare metal atoms only bonded to other metal atoms as well as m ligands bearing m metal atoms. However, usually n>m and thus the number of metal-metal interactions exceeds the number of metal-ligand interactions. These thiolate molecules not only protect the clusters from environment but also allow for reaching atomically precise composition and structures. This wet chemistry route has opened the way to produce gram scale synthesis of thiolate-protected *nanoclusters* with atomic precision [67,68]. So far, more than 100 single-crystal X-ray structures have been reported for the ligand-protected gold nanoclusters [69]. As an illustration, Table 1 summarizes the stoichiometry of 48 thiolate-protected gold nanoclusters reported from the literature [70]. Clearly only specific (m,n) values are observed for nanoclusters. For instance, Au_25_SR_18_^−^ nanoclusters was found to be one of the most stable thiolate-protected nanoclusters reported so far. The crystal structure shows that this nanocluster is composed by a core (bare metal atoms only bonded to other metal atoms) composed of a Au_13_ icosahedron capped by six Au_2_SR_3_ staple-like elements.

**Table 1.** Summary of 48 Gold Nanoclusters from the Experimental Synthesis [70]. The size of gold nanoclusters changes from the smallest cluster Au_10_(SR)_10_ to the largest cluster Au_279_(SR)_84_ and the stabilization ligands are 4-tert-butylbenzenethiolate (S-Ph-t-Bu, TBBT), adamantanethiolate (S-Adm), cyclohexanethiolate (S-c-C_6_H_11_, CHT), 2-phenylethanethiolate (SC_2_H_4_Ph, PET), para-mercaptobenzoic acid (SPh-p-COOH, p-MBA), o-methyl benzenethiol (SPh-o- CH_3_, o-MBT), m-methyl benzenethiol (SPh-m-CH_3_, m-MBT), 2,4-dimethylbenzenethiolate (2,4-DMBT), 3,5-dimethylbenze- nethiolate (3,5-DMBT), phenylthiolate (SPh), cyclopentane- thiolato (S-c-C_5_H_9_), 4-methylbenzenethiolate (p-MBT), and glutathione (SG).

Molecular formula	Protecting thiolate ligands	Electron count (N-M-q)	Molecular formula	Protecting thiolate ligands	Electron count (N-M-q)
Au_10_(SR)_10_	SG	0	Au_24_(SR)_20_	SCH2Ph-t-Bu	4
Au_20_(SR)_16_	TBBT	4	Au_28_(SR)_22_	SCH2Ph-t-Bu	6
Au_18_(SR)_14_	CHT	4	Au_16_(SR)_12_	S-Adm	4
Au_32_(SR)_24_	S-Adm	8	Au_22_(SR)_16_	S-Adm	6
Au_25_(SR)_18_^−^	PET	8	Au_21_(SR)_15_^−^	S-t-Bu	7
Au_21_(SR)_15_^−^	S-Adm	7	Au_28_(SR)_20_^−^	CHT	9
Au_28_(SR)_20_^−^	TBBT	9	Au_23_(SR)_16_^−^	CHT	8
Au_24_(SR)_16_	S-Adm	8	Au_36_(SR)_24_^−^	TBBT/S-c-C5H9,S-Ph, 3,5-DMBT	13
Au_36_(SR)_24_-	3,5-DMBT	13	Au_29_(SR)_19_	S-Adm	10
Au_34_(SR)_22_	CHT	12	Au_44_(SR)_28_	TBBT	16
Au_38_(SR)_24_^−^	PET	15	Au_38_(SR)_24_^−^	PET	15
Au_37_(SR)_23_	CHT	14	Au_42_(SR)_26_^−^	TBBT	17
Au_42_(SR)_26_^−^	TBBT	17	Au_52_(SR)_32_^−^	PET	21
Au_52_(SR)_32_^−^	TBBT	21	Au_56_(SR)_34_	TBBT	22
Au_30_(SR)_18_^−^	S-t-Bu/S-Adm	23	Au_30_(SR)_18_^−^	S-Adm	13
Au_40_(SR)_24_	o-MBT	16	Au_44_(SR)_26_	2,4-DMBT	18
Au_48_(SR)_28_	TBBT	20	Au_43_(SR)_25_	CHT	18
Au_46_(SR)_26_	m-MBT	20	Au_49_(SR)_27_	2,4-DMBT	22
Au_48_(SR)_26_^−^	CHT	23	Au_48_(SR)_26_^−^	m-MBT	23
Au_67_(SR)_35_	SC2H4Ph	32	Au_92_(SR)_44_	TBBT	48
Au_102_(SR)_44_	p-MBA	58	Au_144_(SR)_60_	SCH2Ph	84
Au_133_(SR)_52_	TBBT	80	Au_146_(SR)_57_	p-MBA	89
Au_130_(SR)_50_	p-MBT	80	Au_191_(SR)_66_	TBBT	125
Au_246_(SR)_80_	p-MBT	166	Au_279_(SR)_84_	TBBT	195

**Figure 5.**
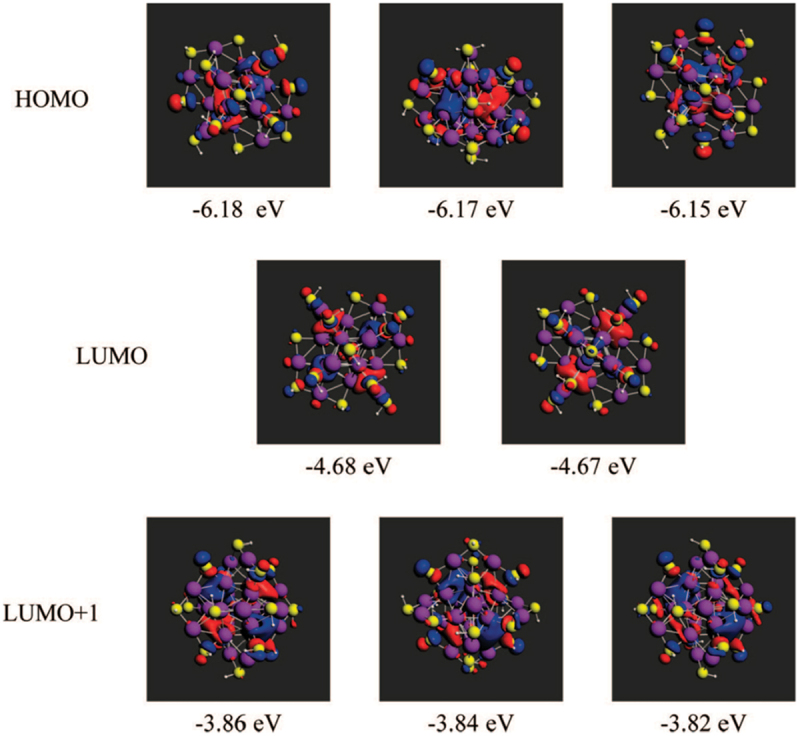
Kohn−sham orbitals and orbital energies for Au_25_(SH)_18_^−^. Reproduced with permission from ref [78]. Copyright 2008 American chemical society.

Derived from the observation of these ‘magic sizes’, thiolate-stabilized gold clusters have been conceptualized within the divide-and-protect theory which has been proved to be very effective in studying their structural characteristics [23]. The gold atoms in the cluster Au_n_(SR)_m_ are in two distinct chemical states (Au_n-m_(AuSR)_m_): metallic (charge-neutral), e.g. Au_n-m_ and oxidized states, e.g. (AuSR)_m_ [71]. With this model, gold nanoclusters form from maximizing Au−Au and Au−S interactions [71]. In its native form, Au_25_SR_18_ bears a negative charge [72–74]. The high stability of this cluster is obtained by its structural robustness and by an electronic stabilization, e.g. an electronic eight-electron shell of delocalized electrons. Au_25_SR_18_^−^ thus present both closed geometric shell (magic number 13) and with eight electrons also a closed electronic shell, which means double stabilization. This electronic stabilization is at the basis of the superatom model introduced into cluster science, referring to clusters whose electronic and chemical properties are approximate to that of an atom [23,75,76]. The stability of superatoms may be understood in most cases within the jellium model, where electrons are confined in a spherically symmetric potential, the electron progressively filling the degenerate electronic levels. To explain the stability of thiolate-protected nanoclusters, Häkkinen and co-workers extended the superatom concept by accounting for ligand interactions with the metallic core [77]. For thiolate-protected metal clusters, there is thus a simple electron count rule, where the electron count is determined as *n* = N − M – q where N is the number of valence orbitals contributed by the metal atoms, M the number of ligands that withdraw one electron (total number of ligands = M + number of Lewis base type ligand) and q the net charge of the complex [23]. While the stability of protected clusters is in first approximation governed by the geometric motif allowing protection for the core by the ligands and bounds energies, if n corresponds to a magic number (*n* = 8 for negatively charged Au_25_SR_18_), the superatom complex may be particularly stable. Looking at molecular orbitals of Au_25_SR_18_^−^, Akola et al. using the mathematical projection techniques and Aikens representing Kohn−Sham orbitals (see Figure 5) [79–81], showed that the HOMO and LUMO orbitals could be considered to be P-like and D-like orbitals, (by analogy with the usual spherical harmonics for p and d functions in atoms). This has some consequences on optical spectra since the first two peaks in the absorption spectrum [81] at ~1.5 eV and ~2.6 eV arise primarily from transitions from the occupied P orbitals into the first and second sets of D orbitals [78].

Nevertheless, if we compare the absorption spectrum of Au_8_^35^ with that of Au_25_SR_18_^36^, both spectra are not exactly the same and the width of the resonances are very different, much broader for Au_25_SR_18._This is probably due to the vibronic coupling with the ligands.

What is striking looking at Table 1 is that there are quite few magic numbers. Clusters with ‘Electron count’ equal to 8, 18, 20, 58 are observed while no clusters are observed for 34, 92, 138. And there are many other electron numbers. This shows that ligands and geometry play an important role. We must not forget that the electronic magic numbers are valid in spherical geometry.

**Figure 6.**
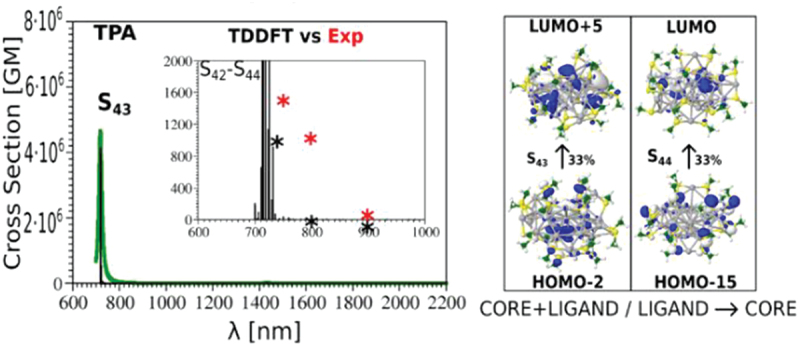
Time-dependent density-functional theory (TDDFT) two-photon absorption spectrum of Ag_31_(SCH_3_)_19_ nanoclusters for the lowest energy structure, plotted in the Goeppert-Mayer units (1 GM = 10^−50^ cm^4^ seconds). Red asterisks label experimental values, while the black ones label theoretical values. Leading excitations responsible for one-photon (OPA) and two-photon (TPA) absorption cross-sections illustrating the participation of the ligands and the core are also shown.

The superatom concept has been successful in explaining the electronic (and therefore optical and catalytic properties) of several magic-number nanoclusters (with the most successful example with Au_25_SR_18_^−^), but this theory presents some weaknesses for describing the thermodynamic stability of thiolate-protected metal nanoclusters in general. First although Au_25_SR_18_^−^ fits well the superatom concept, subsequently, the other charge states (0 and + 1) of the Au_25_SR_18_ nanocluster were obtained via a redox reaction and the crystal structure of the [Au_25_SR_18_]^0^ nanocluster (seven electrons according to electron counting rule) has been found to be very similar to that of the [Au_25_SR_18_]^−1^ nanocluster (eight electrons according to electron counting rule). Also, some ‘magic sizes’ have been successfully experimentally synthesized and isolated under thermodynamic conditions (for instance, Au_20_SR_16_ [82] and Au_36_SR_24_ [83]) that do not fall in the predictions of the superatom theory. The crystal structure also shows that cluster core may be non-spherical requiring to develop new models such as ‘superatomic molecules’ and ‘superatom network’—which have been put forth in an attempt to explain the electronic structure of some stable Au_n_(SR)_m_ nanocluster. ‘superatomic molecules’ can be defined as two superatoms directly bonded while retaining the original morphology [84,85], while superatomic network can be defined as two superatoms stapled by gold-thiolate oligomers [86]. In addition, since this theory is originally derived from metal clusters under the jellium concept (electron counting and shell closure rules), it should be universal for all metal nanoclusters in particular to noble metals that fall on the same column of the periodic table. Yet, silver (or copper) do not form NCs of the same size (number of metal atoms and ligands) and structure [87,88]. For instance using glutathione as ligand molecule, Ag_31_SG_19_/Ag_32_SG_19_, Ag_15_SG_11_, Ag_11_SG_7_ were reported as most stable silver nanoclusters [89,90], while in the same size range, Au_15_SG_13_, Au_18_SG_14_ and Au_25_SG_18_ are observed for gold NCs [91,92]. In this context, composition (Metal versus S content) in addition to NC size and shape (morphology) should be considered to define structural and stability trends. Indeed, the interface structures of thiolated Ag clusters differ significantly from those of thiolated Au clusters. This is mainly due to differences in binding energies of gold- and silver-thiolate motifs, as demonstrated by Aikens et al. [93] The thermodynamic stability model proposed in 2017 by Mpourmpakis and Taylor provides a new exploration of thermodynamic stability of ligand-protected noble metal nanoclusters [94]. In this model, the major factor driving nanocluster stabilization results in a fine energy balance between the core cohesive energy and the shell-to-core binding energy (outlining the importance of the ligand-metal interface). In 2022, Yong Pei and co-corworkers further refine the thermodynamic theory by correlating the thermodynamic stability of thiolate-protected gold clusters with their atomic-level structures and ligand stabilization effects [70]. Protecting ligands thus play a role in structural and electronic of metal nanoclusters, and also affect other properties, such as optical properties. If one plots the optical energy gaps of the reported Au_*n*_(SR)_*m*_ nanoclusters as a function of gold core size (*n*), one sees the general trend of shrinking gap with the increasing size [69]. The energy gap of Au_25_SR_18_^−^ is ∼1.3 eV [69]. Small optical gaps and protection provided by surface thiolated ligands result in efficient near-infrared photoluminescence, which is appealing for bio-applications [95]. Once again, looking at the ‘gold standard’ Au_25_SR_18_^−^ nanoclusters, Aikens et al. have shown that after the sixth excited states, both core-to-core transitions and core-to-semiring transitions are characteristics of frontier orbitals, outlining the importance of the metal-sulfur interface for optical properties [96]. While the contribution of core-to-semiring transitions has a moderate effect on the linear optical properties, Antoine and Bonacic-Koutecky have shown that the metal-sulfur interface and the ‘ligand-core’ contributions are the driving forces for nonlinear optical properties (in particular for two-photon absorption (TPA) cross-section) [97–100]. Such ligand-core contributions (ligand-to-core or inversely core-to-ligand excitations) illustrated in the frontier orbital displayed in Figure 6 are responsible for large transition dipole moments leading to large TPA cross-sections.

## 4. Conclusion

The addition of a protective monolayer stabilizes the exceptional properties of metal clusters. As a result, atomically precise nanoparticles can be produced at the gram scale [101,102]. In particular, for metallic clusters of more than 100 atoms, the properties of the metallic core are well stabilized. These clusters can be functionalized with various ligands for applications in medicine, optical labelling, etc. However, beyond the metallic core, the ‘protecting’ metal-sulfur shell opens the route to new properties and opportunities, in particular for smaller clusters. This is demonstrated in optical properties, in particular in chiroptical [103,104] and nonlinear optics [100]. This may be true also in catalysis and magnetism [105,106].

Small optical gaps can be obtained leading to infrared photoluminescence. The origin of the photoluminescence of noble metal nanoclusters is a controversial topic that has been addressed in many studies but could not be clarified to date [107]. The primary drawback is that the current state-of-the-art chemical synthesis methods only give access to ligand-capped nanoclusters. The ligands capping the metal kernel are involved in luminescence (through ligand/metal charge transfer) and are often sensitive to the environment, which leads to strong variations on the observed photoluminescence. Recently, the generation of ligand-free gold nanoclusters has been demonstrated through a laser-based synthesis method, involving laser fragmentation of pristine laser-generated colloids, followed by a purification procedure. It appears that the emission behavior of small (2–2.5 nm) and ultrasmall (<1 nm, fully characterized by mass spectrometry) NCs is significantly different and dominated by either core- or surface-based emission states. The photoluminescence intensity is a direct function of the surface charge density easily adjusted by the pH of the surrounding medium [108].

Not forget atomic clusters that have proven to be ideal model systems for light-matter interaction studies in all wavelength regimes, being size scalable, easy-to-produce gas phase targets with a simple structure. With the new generation of free electron lasers (FELs), single cluster imaging and simultaneous ion spectroscopy are now possible and diffraction pattern can be extracted from the scattering images. Large xenon clusters up to micron radius were generated. For the first time, their single cluster scattering images were analyzed for cluster morphology and traces of the ultrafast plasma built-up during the femtosecond FEL pulse. The simultaneously measured single cluster ion spectra yield unprecedented insight into the ion dynamics following the interaction. The results will feed both future experimental effort and theoretical modeling [109].

In conclusion, free clusters have been and will continue to be used to get a deep understanding of light matter interactions in finite systems while protected clusters, in parallel to new concepts, allow to take advantage of finite size effects in applications.
